# Clinicopathological Characteristics of Colorectal Neoplasia in Adults Younger than 50 Years: A Real-World Single-Center Study

**DOI:** 10.3390/jcm15166180

**Published:** 2026-08-10

**Authors:** Selcuk Candan, Okan Kati, Oguz Kagan Bakkaloglu, Tugce Eskazan, Ali İbrahim Hatemi, Ahmet Merih Dobrucalı, Billur Canbakan

**Affiliations:** Department of Gastroenterology, Cerrahpaşa Faculty of Medicine, Istanbul University-Cerrahpaşa, 34098 Istanbul, Türkiye; okankati@gmail.com (O.K.); o.k.bakkaloglu@gmail.com (O.K.B.); drtugcecaliskan@gmail.com (T.E.); ihatemi@yahoo.com (A.İ.H.); adobrucali@yahoo.com (A.M.D.); billurcanbakan@yahoo.com (B.C.)

**Keywords:** early-onset colorectal cancer, colorectal neoplasia, advanced adenoma, sessile serrated lesion, colonoscopy

## Abstract

**Background:** Colorectal neoplasia in adults younger than 50 years has attracted increasing clinical attention because of the rising incidence of early-onset colorectal cancer. However, the clinicopathological characteristics of colorectal lesions detected in younger adults remain incompletely described. This study aimed to characterize the clinical, anatomical, and histopathological features of colorectal neoplasia in adults younger than 50 years undergoing colonoscopy. **Methods:** This retrospective single-center study included adults aged 18–49 years who underwent complete colonoscopy between January 2018 and December 2023. After exclusion of individuals with normal colonoscopy findings, 152 patients with at least one colorectal lesion were included. Lesions were classified as benign, advanced, or malignant according to established histopathological criteria. Demographic, endoscopic, and pathological characteristics were analyzed and compared across lesion categories. **Results:** Among 152 patients, 83 (54.6%) had benign lesions, 64 (42.1%) had advanced neoplasia, and 5 (3.3%) had malignant lesions. Advanced lesions were observed predominantly in individuals aged 40–49 years. Most lesions were detected in symptomatic patients undergoing clinically indicated colonoscopy. Histopathological evaluation demonstrated a higher frequency of villous/tubulovillous adenomas, sessile serrated lesions, and high-grade dysplasia among advanced lesions. Larger lesion size was strongly associated with advanced pathological features (*p* < 0.001). In multivariable analysis, patients aged 30–39 years had significantly lower odds of advanced or malignant neoplasia than those aged 40–49 years, whereas no independent associations were observed for sex, smoking status, alcohol use, family history of colorectal cancer, or lesion location. **Conclusions:** In this selected cohort of adults younger than 50 years with detected colorectal lesions undergoing clinically indicated colonoscopy, advanced neoplasia represented a substantial proportion of cases. These findings should not be extrapolated to the general population of adults younger than 50 years or to screening cohorts.

## 1. Introduction

Colorectal cancer (CRC) remains a major global health burden and continues to be one of the leading causes of cancer-related morbidity and mortality [1]. Although the incidence of CRC has declined in older adults—largely due to long-standing screening programs and improved preventive strategies—an opposite trend has emerged in individuals younger than 50 years [2]. This increase in early-onset colorectal cancer (EO-CRC) has been consistently reported across different regions of the world, raising concerns regarding gaps in prevention, delayed diagnosis, and potential shifts in disease biology [3].

The reasons behind the rising rates of EO-CRC are complex and not yet fully clarified. Lifestyle and environmental factors, including Westernized dietary habits, reduced physical activity, obesity, early-life exposures, and alterations of the gut microbiota, have all been implicated [4]. However, these factors alone are insufficient to explain the rapid increase in incidence. Compounding this challenge, adults under 50 generally fall outside established CRC screening algorithms, which may contribute to later-stage presentation and poorer clinical outcomes [5].

Recent studies have suggested that colorectal neoplasia in younger adults may differ from that observed in older populations with respect to anatomical distribution and histopathological characteristics [6]. Several reports have described a greater proportion of proximal lesions and an increasing recognition of sessile serrated lesions among younger individuals [7]. At the same time, advanced pathological features, including larger lesion size, villous architecture, and high-grade dysplasia, appear to become more common with increasing age within the under-50 population [8].

Despite growing interest in early-onset colorectal neoplasia, the clinicopathological spectrum of colorectal lesions detected in younger adults remains incompletely characterized [9]. A better understanding of lesion morphology, anatomical distribution, and histopathological features may improve recognition of clinically significant lesions encountered in routine practice, particularly among symptomatic individuals who fall outside traditional screening programs [10].

Given these emerging patterns, detailed characterization of colorectal polyps in younger adults is essential. Understanding how morphology, localization, and demographic factors intersect in this age group may improve recognition of clinically significant lesions and support diagnostic evaluation, particularly for symptomatic individuals who are not captured by current screening guidelines [11,12].

The present study aimed to provide a real-world clinicopathological characterization of colorectal neoplasia detected in adults younger than 50 years undergoing clinically indicated colonoscopy in a tertiary-care setting. Rather than identifying novel predictors of advanced neoplasia, the study was designed to describe the anatomical, morphological, and histopathological spectrum of lesion-positive young adults encountered in routine clinical practice [13].

In response to the increasing incidence of early-onset colorectal cancer, several international guidelines have lowered the recommended age for average-risk colorectal cancer screening from 50 to 45 years. However, important differences exist between screening colonoscopies performed in asymptomatic average-risk individuals and colonoscopies performed for clinical indications. Symptomatic patients generally represent a selected population with a higher pre-test probability of colorectal pathology and different clinicopathological characteristics. Therefore, findings from clinically indicated colonoscopy cohorts should not be directly extrapolated to screening populations. The present study was designed to characterize the clinicopathological spectrum of colorectal neoplasia in adults younger than 50 years undergoing clinically indicated colonoscopy in routine tertiary-care practice.

## 2. Materials and Methods

This retrospective, single-center study was conducted at a tertiary academic hospital and included all consecutive adults aged 18–49 years who underwent complete colonoscopy at our institution between January 2018 and December 2023. During the study period, all eligible colonoscopy examinations were screened without sampling. A total of 300 colonoscopy examinations met the predefined eligibility criteria and were reviewed. To ensure accurate characterization of neoplastic lesions, individuals with normal colonoscopy findings (*n* = 148) were excluded from the analysis. Thus, the final study cohort consisted of 152 patients with at least one endoscopically detectable colorectal lesion (polyp or malignancy).

### 2.1. Study Population and Eligibility Criteria

All consecutive adults aged 18–49 years with at least one colorectal polyp detected and resected during colonoscopy were eligible for inclusion. Exclusion criteria consisted of incomplete colonoscopy, inadequate bowel preparation, known inflammatory bowel disease, hereditary colorectal cancer syndromes (e.g., Lynch syndrome or familial adenomatous polyposis), prior colorectal surgery, or missing histopathological data. To explore potential age-related phenotypic differences, patients were stratified into three predefined age groups: 18–29, 30–39, and 40–49 years.

### 2.2. Colonoscopy Procedure and Polyp Assessment

All colonoscopies were performed by experienced endoscopists using high-definition video colonoscopes, with ileal intubation and photographic documentation required for study inclusion. During each procedure, polyp characteristics—including size, morphology, number, and anatomical location—were systematically recorded. Anatomical localization was classified as rectum, left colon (sigmoid and descending colon), or right colon (cecum, ascending colon, hepatic flexure, and transverse colon). To evaluate the proposed proximal shift of colorectal neoplasia in younger adults, lesions were additionally analyzed as proximal (right-sided) or distal (left-sided/rectal).

### 2.3. Histopathological Evaluation

All resected specimens were examined by specialized gastrointestinal pathologists. Histological classification included tubular adenoma, tubulovillous adenoma, villous adenoma, hyperplastic polyp, sessile serrated lesion, mixed-type adenoma, or adenocarcinoma. Given the increasing recognition of sessile serrated lesions in colorectal neoplasia, serrated lesions were evaluated separately. Dysplasia was graded as low- or high-grade based on established criteria. Attention was given to sessile serrated lesions due to their strong proximal predilection and association with advanced neoplasia [14].

### 2.4. Definition of Advanced Neoplasia and Malignancy

Advanced neoplasia was defined in accordance with widely accepted criteria, including lesions measuring ≥10 mm in size, villous or tubulovillous histology, high-grade dysplasia, or the presence of sessile serrated lesions. Malignancy was defined by histopathological confirmation of invasive adenocarcinoma [15]. Patient-level category assignment was based on the most advanced lesion identified during the index colonoscopy. In patients with multiple lesions of differing severity, classification followed a predefined hierarchy: invasive adenocarcinoma > advanced non-malignant neoplasia > benign lesion. Accordingly, patients with at least one malignant lesion were assigned to the malignant group, whereas those without malignancy but with at least one advanced lesion were assigned to the advanced neoplasia group.

### 2.5. Clinical Variables and Covariates

Demographic and clinical data—including age, sex, smoking status, alcohol consumption, and family history of colorectal cancer—were extracted from electronic medical records. Family history referred exclusively to colorectal cancer in first-degree relatives and was recorded as a binary variable (present/absent). The age at diagnosis of affected relatives was not consistently available because of the retrospective nature of the study. Exclusion of hereditary colorectal cancer syndromes was based on review of the available clinical records, as formal genetic evaluation was not routinely performed. These variables were included in subgroup analyses to identify clinical factors associated with advanced or malignant pathology. Colonoscopy indications were extracted from the electronic medical records and categorized as anemia, positive fecal occult blood test, abdominal pain, change in bowel habits, abnormal imaging findings, or other clinical indications. Because the study population consisted of individuals undergoing clinically indicated colonoscopy rather than screening examinations, the cohort primarily represented symptomatic or clinically evaluated patients.

### 2.6. Statistical Analysis

Continuous variables are summarized as mean ± standard deviation or median (interquartile range), as appropriate. Categorical variables are presented as frequencies and percentages. Comparisons between lesion categories (benign, advanced, malignant) were conducted using chi-square or Fisher’s exact test for categorical variables and *t*-test or ANOVA for continuous variables. A multivariable logistic regression model was constructed to evaluate independent clinical factors associated with advanced or malignant colorectal neoplasia. Variables constituting the definition of advanced neoplasia were not entered into the model. Variables included in the multivariable model were analyzed using complete-case analysis. Blank fields represented variables that were not applicable to specific patients rather than true missing data, except for one unavailable localization record. All statistical tests were two-sided, and *p* < 0.05 was considered statistically significant.

### 2.7. Real-World Design Considerations

Because this study was based on routine clinical practice within a tertiary referral center, the findings reflect real-world patterns of colorectal polyp detection and management in young adults. This design allowed the capture of clinically relevant features of polyp biology, localization, and neoplastic progression in symptomatic individuals outside standard screening programs.

## 3. Results

A total of 300 colonoscopy examinations performed in adults aged 18–49 years were initially reviewed. After exclusion of 148 individuals with normal colonoscopy findings, the final study cohort consisted of 152 patients with at least one colorectal lesion. Among these lesions, 83 (54.6%) were classified as benign polyps, 64 (42.1%) met criteria for advanced neoplasia, and 5 (3.3%) were identified as malignant lesions (Table 1). Among the 152 patients with colorectal lesions, the most common indications for colonoscopy were anemia (43 patients, 28.3%), changes in bowel habits (26 patients, 17.1%), positive fecal occult blood testing (24 patients, 15.8%), and abnormal imaging findings (23 patients, 15.1%).

The distribution of neoplastic severity demonstrated an age-related gradient across the cohort. Patients aged 40–49 years represented most of the study population (107/152, 70.4%) and accounted for most advanced lesions (51/64, 79.7%) as well as three of the five malignant cases. In contrast, no malignant lesions were detected among individuals aged 18–29 years, in whom only six advanced lesions were identified.

Histopathological evaluation revealed distinct differences between benign and higher-risk lesions. Benign polyps consisted predominantly of tubular adenomas and hyperplastic polyps. Advanced neoplasia, however, demonstrated a substantially greater proportion of villous and tubulovillous adenomas, sessile serrated lesions, and tubular adenomas measuring ≥10 mm. High-grade dysplasia was observed exclusively in the advanced and malignant groups. Among the five malignant lesions, four were adenocarcinomas, and one was a neuroendocrine tumor (Table 2). Among the 69 patients with advanced or malignant neoplasia, 64 had advanced non-malignant lesions, and five had invasive adenocarcinoma. All malignant lesions occurred in patients aged 40–49 years, with three cancers located in the left colon or rectum and two in the right colon. Given the limited number of malignant cases, separate statistical analyses were not performed.

Advanced lesions were significantly larger than benign lesions (*p* < 0.001). Because lesion size constitutes one of the established criteria used in the definition of advanced neoplasia, this association should be interpreted within the context of the study’s definition.

A multivariable logistic regression model was constructed to evaluate independent clinical factors associated with advanced or malignant colorectal neoplasia. Variables used to define the outcome were not entered into the model. After adjustment for age group, sex, smoking status, alcohol use, family history of colorectal cancer, and lesion location, patients aged 30–39 years had significantly lower odds of advanced or malignant neoplasia than those aged 40–49 years (adjusted OR 0.35, 95% CI 0.14–0.86; *p* = 0.022). No significant independent associations were observed for sex, smoking status, alcohol use, family history of colorectal cancer, or right-sided lesion location (Table 3).

Anatomical distribution varied across lesion categories. Benign polyps were predominantly located in the distal colorectum, particularly within the left colon and rectum, whereas advanced lesions demonstrated a more balanced distribution with relatively greater right-sided involvement. Malignant lesions exhibited a mixed anatomical distribution, including two right-sided cancers. However, after correction of localization coding and standardization of segment classification, anatomical location alone was not independently associated with advanced or malignant pathology (χ^2^ test, *p* = 0.122). Similarly, the distribution of benign lesions across colonic segments did not significantly differ from a uniform distribution (*p* = 0.213).

**Figure 1:**
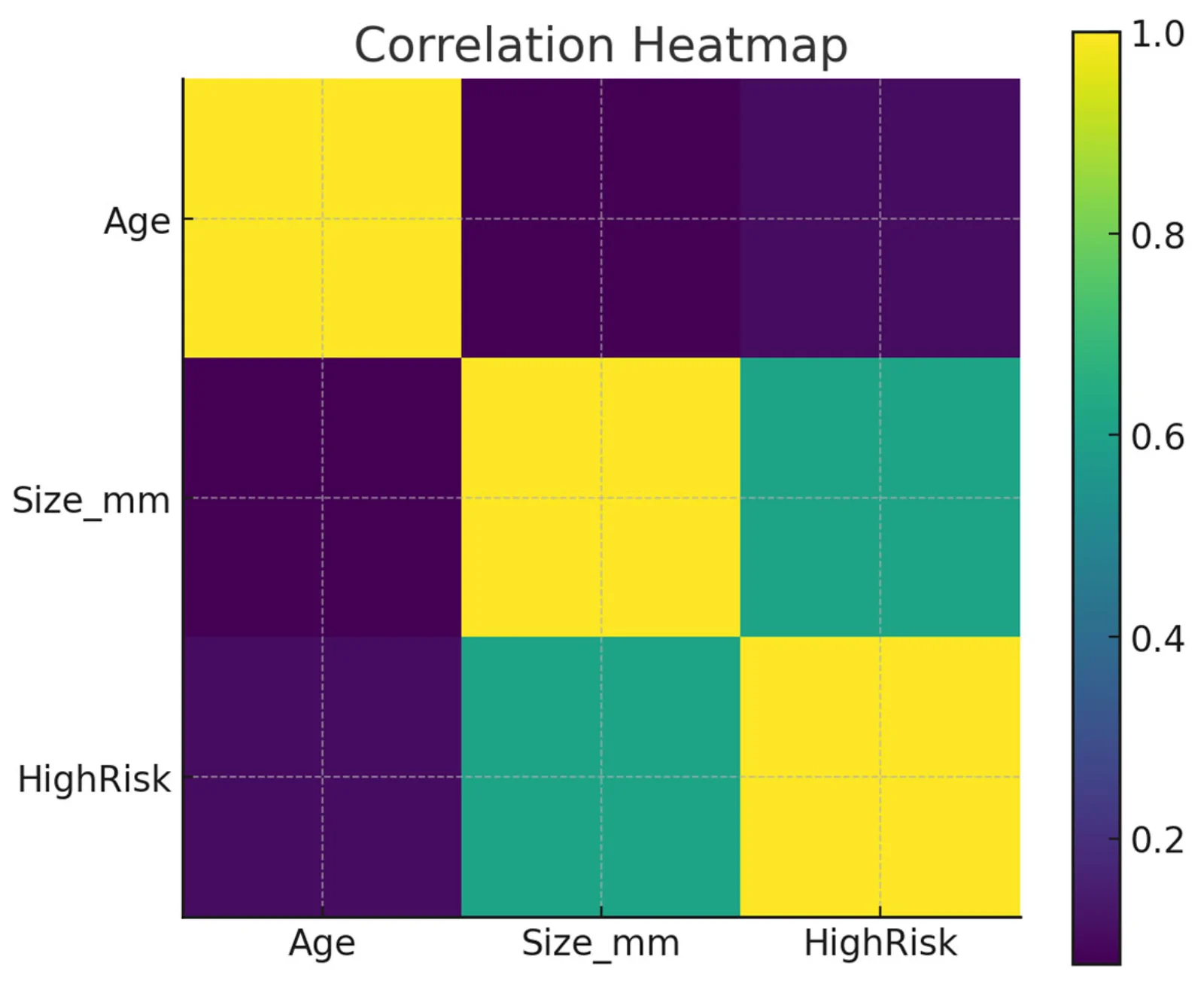
Correlation heatmap illustrating the relationships among age, polyp size, anatomical localization, and neoplastic severity. Polyp size demonstrated the strongest observed correlation with advanced or malignant pathology, whereas age showed a weaker association. Anatomical localization demonstrated limited correlation with neoplastic severity.

Correlation analysis demonstrated a positive association between polyp size and advanced or malignant pathology. Because lesion size constitutes one of the defining criteria for advanced neoplasia, this finding should be interpreted as reflecting the study definition rather than an independent predictive association. Age showed only a weak correlation with neoplastic severity, whereas anatomical localization demonstrated limited correlation (Figure 1).

## 4. Discussion

In this retrospective cohort of adults younger than 50 years with detected colorectal lesions, advanced neoplasia accounted for a substantial proportion of cases [4]. Advanced lesions were observed predominantly in individuals aged 40–49 years and were characterized by larger lesion size, villous/tubulovillous histology, sessile serrated lesions, and high-grade dysplasia. In the revised multivariable analysis, which included only independent clinical variables, patients aged 30–39 years had significantly lower odds of advanced or malignant neoplasia than those aged 40–49 years, whereas no independent associations were observed for sex, smoking status, alcohol use, family history of colorectal cancer, or lesion location. Rather than identifying novel predictors of advanced neoplasia, our findings provide a real-world clinicopathological characterization of lesion-positive adults younger than 50 years undergoing clinically indicated colonoscopy in routine practice [16].

Larger lesion size was more frequently observed among advanced lesions in the present cohort. This observation is consistent with established definitions of advanced colorectal neoplasia, in which lesion size constitutes an important component of risk stratification and has long been recognized as a marker of increased malignant potential [17]. Therefore, the observed association should be interpreted primarily as confirmation of existing pathological criteria rather than as a novel predictive finding. Nevertheless, the high proportion of advanced lesions among larger polyps highlights the continued clinical importance of careful endoscopic assessment, complete resection, and appropriate histopathological evaluation when such lesions are encountered in younger adults [18]. These findings also reinforce previous reports demonstrating that lesion morphology and size remain important indicators of advanced pathology regardless of patient age [19].

Advanced lesions demonstrated a greater frequency of villous/tubulovillous histology, sessile serrated morphology, and high-grade dysplasia compared with benign lesions. These findings are consistent with the established pathological spectrum of advanced colorectal neoplasia and support the concept that histological architecture remains closely linked to neoplastic progression [20]. In particular, the increased representation of sessile serrated lesions among advanced neoplasia is noteworthy, given the growing recognition of serrated lesions as clinically relevant precursors of colorectal cancer [21]. However, because molecular and genetic analyses were not available in the present study, no conclusions can be drawn regarding the specific biological mechanisms or carcinogenic pathways underlying these observations.

Although advanced lesions were observed predominantly in individuals aged 40–49 years, the revised multivariable analysis demonstrated that patients aged 30–39 years had significantly lower odds of advanced or malignant neoplasia compared with those aged 40–49 years [16]. No independent associations were identified for sex, smoking status, alcohol use, family history of colorectal cancer, or lesion location after adjustment. These findings suggest that, within this lesion-positive cohort, increasing age within the under-50 population may be associated with a greater burden of advanced pathology, whereas other routinely available clinical characteristics showed limited discriminatory value [22].

From a clinical perspective, the present findings highlight the diversity of colorectal lesions encountered in adults younger than 50 years undergoing clinically indicated colonoscopy. The relatively high proportion of advanced lesions observed in this selected lesion-positive cohort underscores the importance of careful endoscopic and histopathological evaluation when colorectal lesions are detected in younger individuals. However, these findings reflect a selected symptomatic population and should not be interpreted as prevalence estimates for all adults younger than 50 years or for average-risk screening populations [14,23].

To place our findings into context, Table 4 summarizes recent colonoscopy-based studies evaluating colorectal neoplasia in adults younger than 50 years. Compared with large screening cohorts from the United States and Austria, which reported advanced neoplasia prevalences of approximately 4–5%, our study demonstrated substantially higher rates. This difference is expected because our cohort consisted of clinically indicated, lesion-positive patients rather than asymptomatic individuals undergoing average-risk screening [24,25].

Interestingly, studies conducted in symptomatic Asian and Middle Eastern populations also reported higher lesion yields than screening cohorts, although the prevalence of advanced neoplasia remained considerably lower than that observed in our cohort. These findings further support that differences in patient selection, referral indications, and analytical cohort definitions should be carefully considered when comparing prevalence estimates across studies [26,27].

Several limitations should be acknowledged. First, only patients with at least one detected colorectal lesion were included, thereby precluding estimation of the prevalence of colorectal neoplasia or advanced neoplasia among all adults younger than 50 years undergoing colonoscopy. Consequently, the observed proportion of advanced neoplasia should not be generalized to the broader under-50 population or average-risk screening cohorts. Second, the study was conducted at a single tertiary referral center and primarily included patients undergoing clinically indicated rather than screening colonoscopy, introducing the potential for referral and selection bias. Third, only five malignant lesions were identified, limiting cancer-specific analyses and preventing robust conclusions regarding invasive colorectal cancer. Fourth, molecular and genetic data were not available, precluding assessment of the biological mechanisms underlying early-onset colorectal neoplasia. Additionally, certain potentially relevant clinical variables, including body mass index, diabetes status, and medication use (e.g., aspirin or NSAIDs), were not consistently available because of the retrospective design. Information regarding whether all examinations represented a patient’s first lifetime colonoscopy was also not consistently available. Finally, no comparison group aged 50 years or older was included, limiting direct age-based comparisons.

## 5. Conclusions

In this retrospective cohort of adults younger than 50 years with detected colorectal lesions, advanced neoplasia represented a substantial proportion of cases, particularly among individuals aged 40–49 years. Larger lesion size, villous/tubulovillous histology, and sessile serrated morphology were more frequently observed in advanced neoplasia, whereas anatomical localization showed limited association with neoplastic severity. These findings provide a detailed, real-world clinicopathological characterization of colorectal neoplasia in lesion-positive adults younger than 50 years undergoing clinically indicated colonoscopy and contribute to a better understanding of the lesion spectrum encountered in routine tertiary-care practice. However, because only patients with detected colorectal lesions undergoing clinically indicated colonoscopy were included, these findings should not be generalized to asymptomatic or screening populations.

## Figures and Tables

**Table 1:** Demographic and clinical characteristics of the study population.

Variable	Benign (*n* = 83)	Advanced/Malignant (*n* = 69)	*p* Value
Age, years, median (IQR)	43.0 (37.5–46.0)	45.0 (41.0–48.0)	0.022
Male sex, *n* (%)	45 (54.2)	40 (58.0)	0.764
Current smoking, *n* (%)	11 (13.3)	5 (7.2)	0.349
Alcohol use, *n* (%)	7 (8.4)	11 (16.2)	0.227
Family history of CRC, *n* (%)	25 (30.1)	19 (27.5)	0.865
Polyp size, mm, median (IQR)	4.0 (3.0–6.0)	10.0 (10.0–15.0)	<0.001
Left colon, *n* (%)	38 (46.3)	37 (53.6)	
Rectum, *n* (%)	24 (29.3)	14 (20.3)	0.440
Right colon, *n* (%)	20 (24.4)	18 (26.1)	

Footnote: Values are presented as median (IQR) or *n* (%). Percentages were calculated within each study group unless otherwise specified. Anatomical localization data were available for 82 of 83 patients in the benign group; therefore, localization percentages were calculated using patients with available localization data as the denominator. CRC, colorectal cancer; IQR, interquartile range.

**Table 2:** Histopathological characteristics according to neoplastic category.

Final Histopathological Diagnosis	Benign (*n* = 83), *n* (%)	Advanced/Malignant (*n* = 69), *n* (%)	*p* Value
Tubular adenoma	42 (50.6)	31 (44.9)	0.517
Tubulovillous adenoma	0 (0.0)	19 (27.5)	<0.001
Villous adenoma	0 (0.0)	3 (4.3)	0.091
Hyperplastic polyp	31 (37.3)	2 (2.9)	<0.001
Sessile serrated lesion	0 (0.0)	8 (11.6)	0.001
Other/unspecified lesion	10 (12.0)	1 (1.4)	0.012
Adenocarcinoma	0 (0.0)	4 (5.8)	0.040
Neuroendocrine tumor	0 (0.0)	1 (1.4)	0.454

Footnote: Values are presented as *n* (%). Each patient was classified once according to the final histopathological diagnosis of the most advanced lesion identified during the index colonoscopy. *p* values were calculated using Fisher’s exact test. Percentages were calculated within each neoplastic category.

**Table 3:** Multivariable logistic regression analysis of clinical factors associated with advanced or malignant colorectal neoplasia.

Variable	Adjusted OR (95% CI)	*p* Value
Age 18–29 vs. 40–49 years	1.12 (0.30–4.14)	0.871
Age 30–39 vs. 40–49 years	0.35 (0.14–0.86)	0.022
Male sex	1.05 (0.50–2.17)	0.906
Current smoking	0.30 (0.08–1.06)	0.061
Alcohol use	2.69 (0.82–8.79)	0.102
Family history of CRC	1.27 (0.56–2.84)	0.568
Right-sided lesion location	1.87 (0.90–3.88)	0.095

Dependent variable: Advanced or malignant colorectal neoplasia (*n* = 69) versus benign lesions (*n* = 83). Variables used to define the outcome were not entered into the multivariable model. Reference categories were age 40–49 years, female sex, non-smoker, no alcohol use, no family history of colorectal cancer, and left-sided/rectal lesion location. OR, odds ratio; CI, confidence interval; CRC, colorectal cancer.

**Table 4:** Comparison of recent studies on colorectal neoplasia in adults <50 years.

Study	Population	*n*	Lesion Prevalence *	Adv. Neo. ^†^	CRC	Design
Liang et al., 2022 [24]	Screening	129,736	26.4	4.8	NR ^‡^	National Registry
Penz et al., 2023 [25]	Screening	11,103	10.5	3.9	NR ^‡^	National Registry
Obeidat et al., 2024 [26]	Rectal bleeding	3422	12.5 (polyps)	1.75	1.3	Single-center
Le et al., 2025 [27]	Clinically indicated	1696	13.0	3.7	1.6	Single-center
Present study	All eligible colonoscopies	300 ^§^	50.7	21.3 ^§^	1.7 ^§^	Single-center

Footnotes: * Lesion prevalence was reported according to the original definitions used in the respective studies (e.g., adenoma, colorectal neoplasia, or colorectal polyps). ^†^ Advanced neoplasia was reported according to the original definition adopted in each study. ^‡^ CRC prevalence was not reported separately in the original screening cohort. ^§^ Among all eligible colonoscopies screened (*n* = 300). In the lesion-positive analytical cohort (*n* = 152), advanced neoplasia and CRC accounted for 42.1% and 3.3%, respectively.

## Data Availability

The datasets generated and/or analyzed during the current study are available from the corresponding author upon reasonable request.
